# High copy wildtype human 1N4R tau expression promotes early pathological tauopathy accompanied by cognitive deficits without progressive neurofibrillary degeneration

**DOI:** 10.1186/s40478-015-0210-6

**Published:** 2015-06-04

**Authors:** Jeanna M. Wheeler, Pamela J. McMillan, Michele Hawk, Michiyo Iba, Linda Robinson, George J. Xu, Beth A. Dombroski, Doori Jeong, Marc A. Dichter, Halvor Juul, Elaine Loomis, Murray Raskind, James B. Leverenz, John Q. Trojanowski, Virginia M.Y. Lee, Gerard D. Schellenberg, Brian C. Kraemer

**Affiliations:** Geriatric Research Education and Clinical Center, Veterans Affairs Puget Sound Health Care System, Seattle, WA 98108 USA; Mental Illness Research Education and Clinical Center, Veterans Affairs Puget Sound Health Care System, Seattle, WA 98108 USA; Department of Psychiatry and Behavioral Sciences, University of Washington, Seattle, WA 98195 USA; Department of Pathology and Laboratory Medicine, Perelman School of Medicine, University of Pennsylvania, Philadelphia, PA 19104 USA; Department of Neurology, Perelman School of Medicine, University of Pennsylvania, Philadelphia, PA 19104 USA; Cleveland Clinic Lou Ruvo Center for Brain Health, Cleveland, OH 44195 USA; Division of Gerontology and Geriatric Medicine, Department of Medicine, University of Washington, Seattle, WA 98104 USA

**Keywords:** Tau, Aggregation, Hyperphosphorylation, Tauopathy

## Abstract

**Introduction:**

Accumulation of insoluble conformationally altered hyperphosphorylated tau occurs as part of the pathogenic process in Alzheimer’s disease (AD) and other tauopathies. In most AD subjects, wild-type (WT) tau aggregates and accumulates in neurofibrillary tangles and dystrophic neurites in the brain; however, in some familial tauopathy disorders, mutations in the gene encoding tau cause disease.

**Results:**

We generated a mouse model, Tau4RTg2652, that expresses high levels of normal human tau in neurons resulting in the early stages of tau pathology. In this model, over expression of WT human tau drives pre-tangle pathology in young mice resulting in behavioral deficits. These changes occur at a relatively young age and recapitulate early pre-tangle stages of tau pathology associated with AD and mild cognitive impairment. Several features distinguish the Tau4RTg2652 model of tauopathy from previously described tau transgenic mice. Unlike other mouse models where behavioral and neuropathologic changes are induced by transgenic tau harboring *MAPT* mutations pathogenic for frontotemporal lobar degeneration (FTLD), the mice described here express the normal tau sequence.

**Conclusions:**

Features of Tau4RTg2652 mice distinguishing them from other established wild type tau overexpressing mice include very early phenotypic manifestations, non-progressive tau pathology, abundant pre-tangle and phosphorylated tau, sparse oligomeric tau species, undetectable fibrillar tau pathology, stability of tau transgene copy number/expression, and normal lifespan. These results suggest that Tau4RTg2652 animals may facilitate studies of tauopathy target engagement where WT tau is driving tauopathy phenotypes.

**Electronic supplementary material:**

The online version of this article (doi:10.1186/s40478-015-0210-6) contains supplementary material, which is available to authorized users.

## Introduction

The microtubule binding protein tau can aggregate to form pathological lesions in a variety of dementia disorders including Alzheimer’s disease (AD), frontotemporal lobar degeneration (FTLD), Pick’s disease, corticobasal degeneration, progressive supranuclear palsy, and agyrophilic grain disease. Collectively, neurodegenerative diseases with pathological tau aggregates are called tauopathies. The unifying features of these disorders are tau pathology, neurodegeneration, and an overlapping suite of neurological features including abnormal cognitive function and/or motor disturbance. While mutations in human *MAPT*, the gene encoding tau, promote tau aggregation and drive a subset of familial FTLD with underlying tau neuropathology, the vast majority of tauopathy cases do not have *MAPT* mutations and exhibit aggregation of wildtype (WT) tau.

In AD, WT human tau forms the pathological tau species contributing to neuronal dysfunction and neurodegeneration. Significant efforts have explored the relationship between abnormal tau and disease. For instance, previous studies demonstrated that pathological tau, not amyloid deposition, correlates with the cognitive decline seen in AD [1], and neurofibrillary degeneration coincides with neuronal loss in AD [2]. Previous work suggested that accumulation of pre-tangle conformations of tau drive progression in AD [3, 4]. However, the exact nature of the toxic species of pathological tau remains a topic of ongoing debate [5–8].

To model sporadic tauopathy we overexpressed the most abundant isoform of WT human tau (1N4R) to provoke pathological changes associated with tauopathy. We generated a new mouse model with abundant overexpression of a normal human tau cDNA sequence that exhibits abundant pre-tangle tau neuropathology accompanied by behavioral abnormalities. While many mouse models of tauopathy have been generated, ours is distinguished by the robustness, rapid onset, and relative stability of the phenotype over time. In particular, the model may have utility for studies of tau transmission as reported by Clavaguera et al [9] as well as for pre-clinical studies where the ability to assess target engagement at early time points preceding neurofibrillary degeneration is desirable, thus avoiding the time and cost involved in characterizing aged mice.

## Materials and methods

### Antibodies

MC1 and Alz50 (provided by Peter Davies, Albert Einstein College) are conformation specific mouse monoclonal antibodies that recognize amino acids 7–9 and 313–322 (MC1) [10] or amino acids 5–15 and 312–322 (Alz50) [11] of tau and are specific for pathological tau. AT8 (Thermo Scientific, Rockford, IL) is a phosphorylation-dependent mouse monoclonal antibody that recognizes PHF-tau phosphorylated on dual sites Ser202 and Thr205. Other antibodies used in this study that recognize phosphorylated epitopes of tau include AT180 (pThr231; Thermo Scientific), AT270 (pThr181; Innogenetics), and PHF1 (pSer396/pSer404; provided by Peter Davies) [12]. Antibody TOC1 (tau oligomeric complex 1), which selectively labels tau dimers and oligomers, but does not label filaments [13] was kindly provided by Lester Binder (Michigan State University). Rabbit polyclonal 17025 is a pan-tau antibody recognizing total mouse and human tau raised against full length recombinant tau [14]. SMI31 (Covance, Princeton, N.J.) is a mouse monoclonal antibody that reacts with phosphorylated neurofilament H. The anti-actin mouse monoclonal antibody was obtained from the developmental studies hybridoma bank (dshb.biology.uiowa.edu).

### Construction of Transgenic mice

[B6.Cg-Tg(Thy1-MAPT*)2652Gds]. The cDNA encoding the most abundant brain isoform (1N4R) of tau was cloned into the unique XHO I site in a mouse neuron specific expression vector, pThy1.2 [15]. Transgenic (Tg) mice were generated by pronuclear microinjection of the Thy1.2::Tau (1N4R) transgene at the University of Washington Nathan Shock Center Transgenic Animal Model Core (Warren Ladiges, PI). Founders were identified by PCR analysis of tail biopsies as described below. Founder mice were intercrossed with C57BL/6J mice to establish lines. The 2652 line was the focus of characterization due to its high level tau expression and robust phenotype. Mice from the Tau4RTg2652 line used in these studies were backcrossed 6–9 generations (incipient congenic) with the C57BL/6J strain. This mouse strain has been deposited with the Mutant Mouse Regional Resource Centers (MMRRC) and is available under the stock number MMRRC:036717 and the strain name of B6.Cg-Tg(Thy1-MAPT*)2652Gds.

### Genotyping

Mice were genotyped using DNA prepared from tail clips from live mice. The presence of the Thy1.2::Tau (1N4R) transgene was detected by PCR analysis using primers yielding a 316 bp product for hemizygous Tg mice and no product for WT animals: cDNA trunc-F, AAGATCGGCTCCACTGAGAA; cDNA trunc-R, GGACGTGGGTGATATTGTCC.

### Copy Number analysis

A custom TaqMan® copy number assay was designed by Applied Biosystems® (Assay #MAPT_CCRR9BJ). Primers are within exon 9 of *MAPT:* Forward primer sequence ACCCAAGTCGCCGTCTTC; reverse primer sequence CCGATCTTGGACTTGACATTCTTCA; Reporter sequence: CAGACAGCCCCCGTGCCCA. The TaqMan® Copy Number Reference Assay, Mouse *Tert* (part # 4458368) was used for an endogenous control. DNA was extracted from mouse tail samples using DNeasy ™ Blood and Tissue Kit (Qiagen). DNA was quantitated using the Nanodrop 2000 spectrophotometer (Thermo Fisher Scientific), and DNA samples were diluted in water to a concentration of 1 ng/μL (to provide 4ng of DNA/reaction). This is less than the manufacturer’s recommended 5 ng/μ L, because of the exceptionally high copy number of the target DNA. TaqMan® Universal PCR Master Mix (Applied Biosystems) was used, in a 96 well plate format, according to the supplier’s protocol for TaqMan® Copy Number Assays; with the exception that primers for the reference assay (*Tert*) were not placed in the same reaction well as those for Exon 9, the target sequence. Primers failed to amplify *Tert* when duplexed in the same reaction with primers for the target, likely due to the overabundance of the target sequence relative to the reference sequence. Samples were run in quadruplicate. DNA from a single mouse from a different Tg mouse line [B6.Cg-Mapt^tm1Hnd^Tg(MAPT*)1Gds] was used as a calibrator sample for all assay plates. This mouse line has a low copy number of the human *MAPT* transgene. Reactions were run on a 7900HT Fast Real-Time PCR System instrument, using the amplification program: 50 °C for 2 min, 95 °C for 10 min, 95 °C for 15 s and 60 °C for 60 s (the last 2 steps repeated 40 times). Generation of standard curves showed that both *Tert* and *MAPT* PCR reactions approached 100 % efficiency and were within the linear range using 4 ng of DNA from Tau4RTg2652 mice. Results were analyzed using a C_T_ threshold of 0.2. Because reference and target amplification reactions were performed in different wells, the comparative C_T_ method (∆∆C_T_) of analysis was used to calculate copy number. In order to obtain an estimation of the absolute copy number of the *MAPT* cDNA transgene, the ∆ CT between *Tert* and *MAPT* reactions was calculated.

### Behavioral analysis

All mice were bred and housed at the University of Pennsylvania (Penn) animal facility, and all experiments were approved by the IACUC committee. Mice were housed on a 12:12 light cycle in static microisolators cages, with rodent chow and water available *ad libitum*. Nestlets (Animal Specialties and Provisions, Quakertown, PA) were added to cages for environmental enrichment in accordance with Penn IACUC mouse care guidelines. All behavioral experiments were conducted during the light phase. Subjects were male and female Tau4RTg2652 animals and their non-Tg littermates. Two separate groups of animals were tested at each of three ages, to eliminate the potential effects of repeated testing. The group sizes were: 3 month group A, 14 Tg and 11 WT; 3 month group B, 12 Tg and 12 WT; 6 month group A, 9 Tg and 11 WT; 6 month group B, 10 Tg and 10 WT; 11 month group A, 7 Tg and 8 WT.

Open field testing was conducted in a 16 × 16 inch square clear plastic arena. All animals were moved into the testing room 30–40 min prior to start of testing. The arena was cleaned with 70% ethanol between animals. Total activity, vertical rearing, and activity in the center of the arena were measured for 10 min using a San Diego Instruments Photobeam Activity System.

The Barnes maze (San Diego Instruments) is a circular white plastic platform 36 inches in diameter with 20 holes around the perimeter. Nineteen holes were occluded with shallow, off-target boxes and one hole leading to a target escape box. Latency to enter the escape box was recorded. Placards with unique monochromatic patterns (vertical lines, horizontal lines, large circles, etc.) were placed around the room to be used as landmarks to find the escape box. The placards were approximately 12" by 14" and were placed at various heights. In addition to these placards a stuffed frog, a tether ball, and a car license plate were also suspended in various areas of the room. The light level on the platform was 375 lux. Mice are placed in a box in the center of the maze for 30 s at the beginning of each trial, so the direction each mouse faces at the trial start is random. When the start box is removed, each animal has a maximum of 2 min to locate the escape box. If the animal locates the hole, it is allowed to shelter there for 1 min before being returned to the home cage. If the animal fails the task, it is gently guided to the escape box by the investigator and allowed to shelter there for 1 min. All animals were moved into the testing room 30–40 min prior to start of testing, and the maze was cleaned with 70% ethanol between animals. Training trials were conducted twice daily for four consecutive days.

Two tests of grip strength were used: ability to hang from an inverted wire grid, and ability to grip a wire bar when pulled by the tail. For the inverted grid test, animals were placed onto a standard wire cage top and encouraged to grip it by gently shaking it. Then the wire top was slowly inverted and suspended approximately 30cm over a padded surface. The amount of time each animal was able to grip the wire grid was recorded, up to a maximum of 2 min. Each animal received three trials on the same day, with a minimum of 30 min rest between trials. Data presented are the average of the three trials for each animal. The T-bar pull-test was performed using a IITC Life Science Grip Strength Meter (Woodland Hills, CA) force gauge with a metal T-bar attached. Animals were held by the tail and allowed to grip the T-bar with forelimbs only, then gently pulled away from the grid to measure maximum grip strength. Each animal received five trials on the same day, with approximately 20 min rest between trials. Data are the average of the five trials for each animal.

Rotorod testing was conducted using an accelerating UGO Basile Rota Rod. The rod surface is covered with ridged plastic, located 16cm above the floor. All mice were transferred to the testing room 30–40 min prior to the start of testing, and were tested on four consecutive days. On the first three days, all mice received three training trials separated by approximately 35 min rest time. For all training trials, mice were placed onto a slowly moving rotorod (3 RPM). Once all mice in a testing group were on the rotorod, the apparatus was accelerated up to 16.75 RPM over 2 min. On the fourth day, mice also received three trials (45–50 min of rest between trials), but the rod was accelerated up to 39 RPM over 5.5 min to test maximum motor coordination abilities.

Statistical analysis was performed using Statistica (StatSoft). For all measures, sex and testing group were included as factors in the ANOVA. If either sex or group did not have a significant effect on a particular measure, then the analysis was repeated without that factor. For repeated measures (rotorod and Barnes testing), ANOVA was followed up with planned comparisons of the two genotypes on each testing day.

### Electroencephalographic analysis

Homozygous 4RTauTg3652 mice were anesthetized with isofluorane and implanted bilaterally with electrodes—one with bilateral skull screw electrodes and two with one skull screw and contralateral depth electrodes in the cortex and hippocampus. Following recovery mice were placed in recording cages for 24/7 video EEG recording for 4 to 13 days. EEG was filtered (high pass 0.1 Hz, low pass 600 Hz) and digitized at 2000 Hz.

### Immunoblot analysis

We conducted a sequential extraction of tau protein using buffers of increasing solubilizing strength as previously described [14]. Mouse brains from three non-Tg and nine Tau4RTg2652 mice at varying ages were isolated and snap frozen on liquid nitrogen prior to extraction. Mouse brain hemispheres were homogenized in high salt re-assembly buffer (RAB-High Salt [0.1 M MES, 1 mM EGTA, 0.5 mM MgSO_4_, 0.75 M NaCl, 0.02 M NaF, 0.5 mM PMSF, 0.1 % protease inhibitor cocktail, pH 7.0]) and ultra-centrifuged at 50,000x gravity yielding the soluble fraction (supernatant) and an insoluble pellet. Next, myelin was floated by resuspending the pellet in RAB/1M sucrose and centrifuging as above. To extract detergent soluble tau, the RAB insoluble material was re-extracted with an ionic and non-ionic detergent containing RIPA buffer [50 mM Tris, 150 mM NaCl, 1% NP40, 5mM EDTA, 0.5 % DOC, 0.1 % SDS, 0.5 mM PMSF, 0.1 % protease inhibitor cocktail, pH 8.0] and centrifuged as above yielding abnormal tau in the supernatant. Finally, the detergent insoluble pellet was re-extracted with 70 % Formic Acid (FA) to solubilize detergent insoluble tau. Total protein fractions containing ~15 ug of protein per lane were boiled 5 min and loaded onto 10 % pre-cast SDS-PAGE gels (Biorad, Hercules, CA). Subsequent RIPA and FA extracted fractions were normalized according to the levels of total protein in the total fraction. For semi-quantitative immunoblotting, we detected mouse and human tau using the pan tau antibody 17025 at a dilution of 1:3000 as described previously [16] and anti-actin antibody at 1:1000 (DSHB). Densitometry measurements were performed using Adobe Photoshop to determine total tau levels in non-Tg and Tg mice.

### Immunohistochemistry and histological stains

Tg mice at three months (*n* = 9) and 1 year (*n* = 12) were examined by immunohistochemistry with antibodies MC1 and AT8. A subset of these brains was also immunostained with Alz50, AT180, AT270, PHF-1, TOC1 and SMI31. Mice were anesthetized and fixed by transcardial perfusion with 4% paraformaldehyde. Brains and spinal cords were removed and paraffin embedded for sectioning. Coronal sections from the forebrain, hippocampus, and brainstem were cut at 10 μm thickness and stored at 4 °C until use. Sections were deparaffinized and rehydrated through alcohols, and an antigen retrieval step consisting of heat pretreatment by microwave in citrate buffer was used when necessary. Sections were treated for endogenous peroxidases with 3 % hydrogen peroxide in PBS (pH 7.4), blocked in 5 % non-fat milk in PBS, and incubated with primary antibody overnight at 4°C followed by biotinylated secondary antibody for 45 min at room temperature. Finally, sections were incubated in an avidin-biotin complex (Vector’s Vectastain Elite ABC kit, Burlingame, CA) and the reaction product was visualized with 0.05 % diaminobenzidine (DAB)/0.01% hydrogen peroxide in PBS. Negative controls with secondary antibody alone did not immunostain tissue sections (data not shown). The presence of neurofibrillary tangles (NFTs) was assessed by Gallyas silver and Thioflavin-S staining using standard methods [17]. Bielschowsky silver stain was utilized to assess axonal pathology [18].

### Photomicrography and figure preparation

Photomicrographs were taken with a digital camera and imported into Adobe Photoshop for mounting. To optimize visualization of staining, photomicrographs were modified when necessary by adjusting brightness and contrast.

## Results

**Fig. 1:**
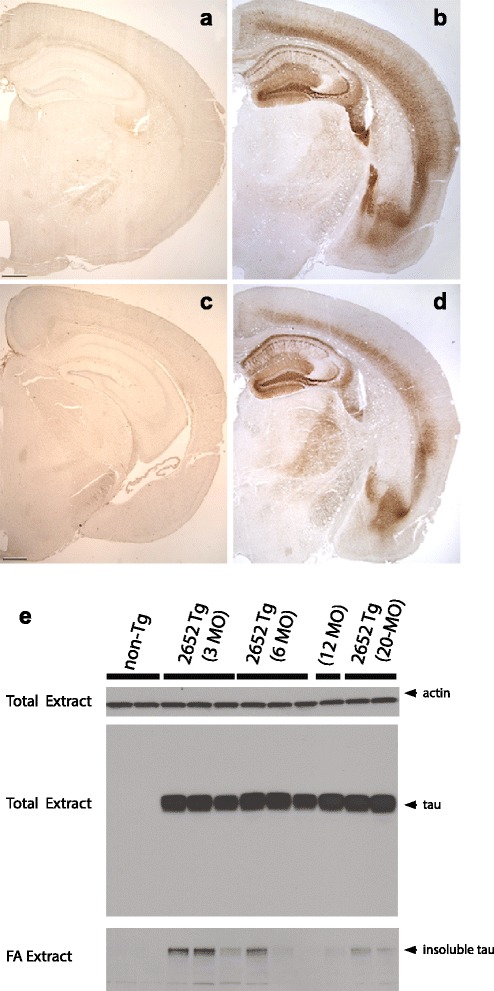
Tau4RTg2652 animals accumulate conformationally altered abnormally phosphorylated pathological tau. Age matched non-Tg mice exhibit no detectable phosphorylated tau by AT8 immunohistochemistry (**a**) as compared to TauTg2652 mice (**b**) which are immunoreactive as early as 3 months of age. Likewise, non-Tg mice exhibit no detectable pre tangle conformations of tau by MC1 immunohistochemistry (**c**) as compared to TauTg2652 mice (**d**) where pre-tangles are evident as early as 3 months of age. Scale bars = 500 μm. (**e**) Immunoblot analysis of sequential extraction of detergent insoluble tau protein was detected with pan-tau antibody 17-25 [14]. Brain hemispheres were homogenized and lysates compared from 3 mice per group (3 month old, 6 month old, 20 month old tau Tg compared with Non-Tg mice). Total extracts were first probed for tau and actin to determine total tau levels relative to loading control. To measure detergent insoluble tau species, mouse brains were subjected to a sequential extraction using buffers of increasing solubilizing strength. Note the FA fraction represents detergent insoluble tau. Results are shown for 3 different nTg mice, and for 3 different Tau4RTg2652 mice for each age group

In Alzheimer’s disease and related tauopathies, pathological tau accumulates in neurons and in some cases glia. To model early tau changes associated with disease progression, we generated Tg mice that overexpress the 1N4R isoform of human tau in mouse neurons using the Thy 1.2 promoter. Among the lines generated, Tau4RTg2652 had the most robust tau expression levels and stable reproducible expression of early tauopathy markers relative to non-Tg mice. Brains from Tau4RTg2652 mice were robustly immunoreactive when immunostained using antibodies associated with tau pathology, while brains from non-transgenic mice showed no immunoreactivity (Fig. 1 a-d). Tau4RTg2652 animals exhibited ~12 fold overexpression of human tau relative to endogenous mouse tau and displayed consistent transmission of approximately 143 ± 20 copies of the transgene (Additional file 1: Figure S1). Tau4RTg2652 mice exhibit consistently high levels of total tau protein (Fig. 1e) and approximately normal lifespan with no obvious cause of early mortality or developmental defects;

### Accumulation of pathological tau

To measure total tau levels, whole mouse brains from 3 month old, 6 month old, 12 month old, and 20 month old mice were homogenized and immunoblotted with a pan-tau specific antibody, demonstrating total human tau levels ~12 fold greater than normal endogenous mouse tau in non-Tg mice (Fig. 1). We homogenized brain hemispheres from non-Tg mice and the aged Tau4RTg2652 animals in RAB, a high salt buffer, yielding the soluble tau fraction. We observe consistently high levels of total tau among all Tg animals in this fraction. We re-extracted material insoluble in RAB with RIPA, an ionic and non-ionic detergent containing buffer yielding the detergent soluble fraction. Subsequently, we recovered detergent insoluble material by extraction with formic acid. Non-Tg animals exhibit nearly all tau in the soluble fraction (RAB) with no detergent insoluble tau detected. In contrast the Tau4RTg2652 mice exhibit marked but highly variable accumulation of aggregated tau in the detergent soluble (RIPA) and detergent insoluble (FA) fractions.

**Fig. 2:**
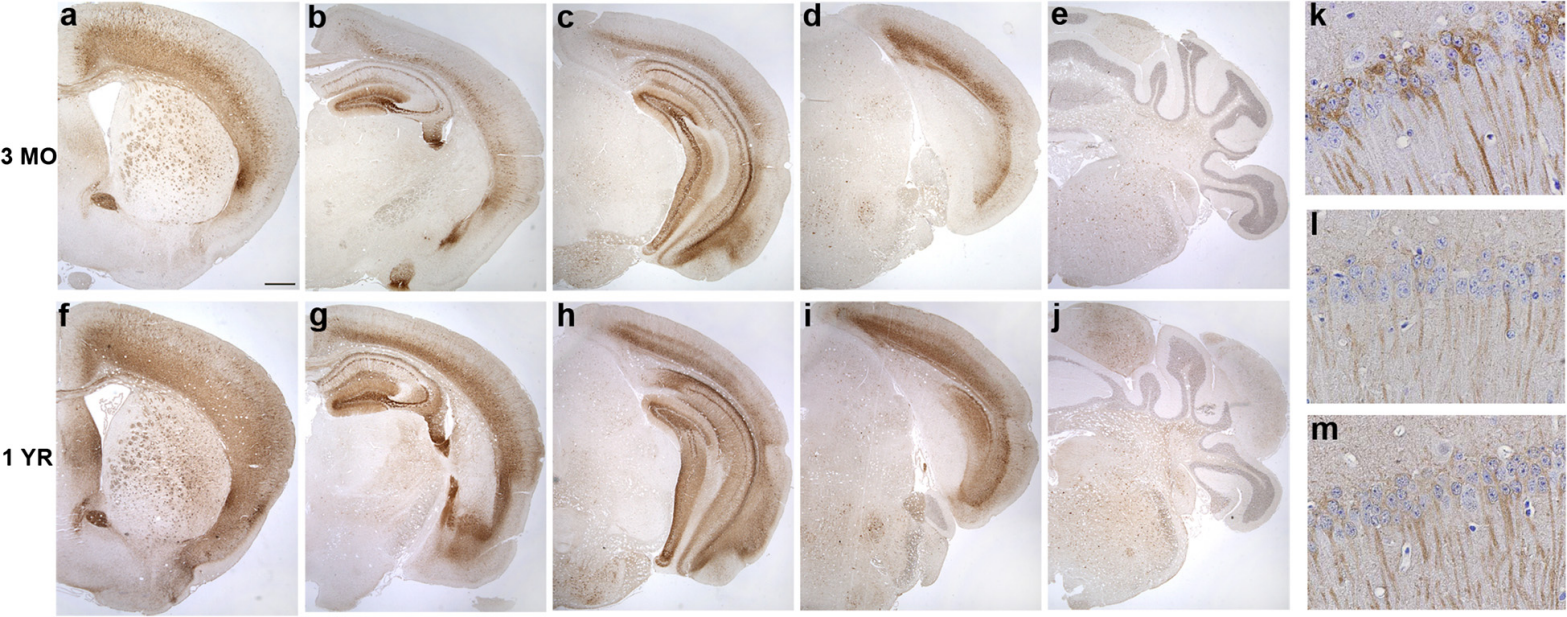
Tau4RTg2652 mice are immunoreactive for a variety of phospho-tau epitopes across multiple brain regions. At three months (**a**-**e**) and one year (**f**-**j**), AT8 immunoreactive neurons are numerous in the forebrain and hippocampus and moderately immunoreactive in the hindbrain. One year old mice demonstrate immunoreactivity to phospho-tau antibodies AT180 (**k**), AT270 (l) and PHF1 (m) in the CA1 region of the hippocampus. Scale bars = 500um (**a**-**j**)

**Fig. 3:**
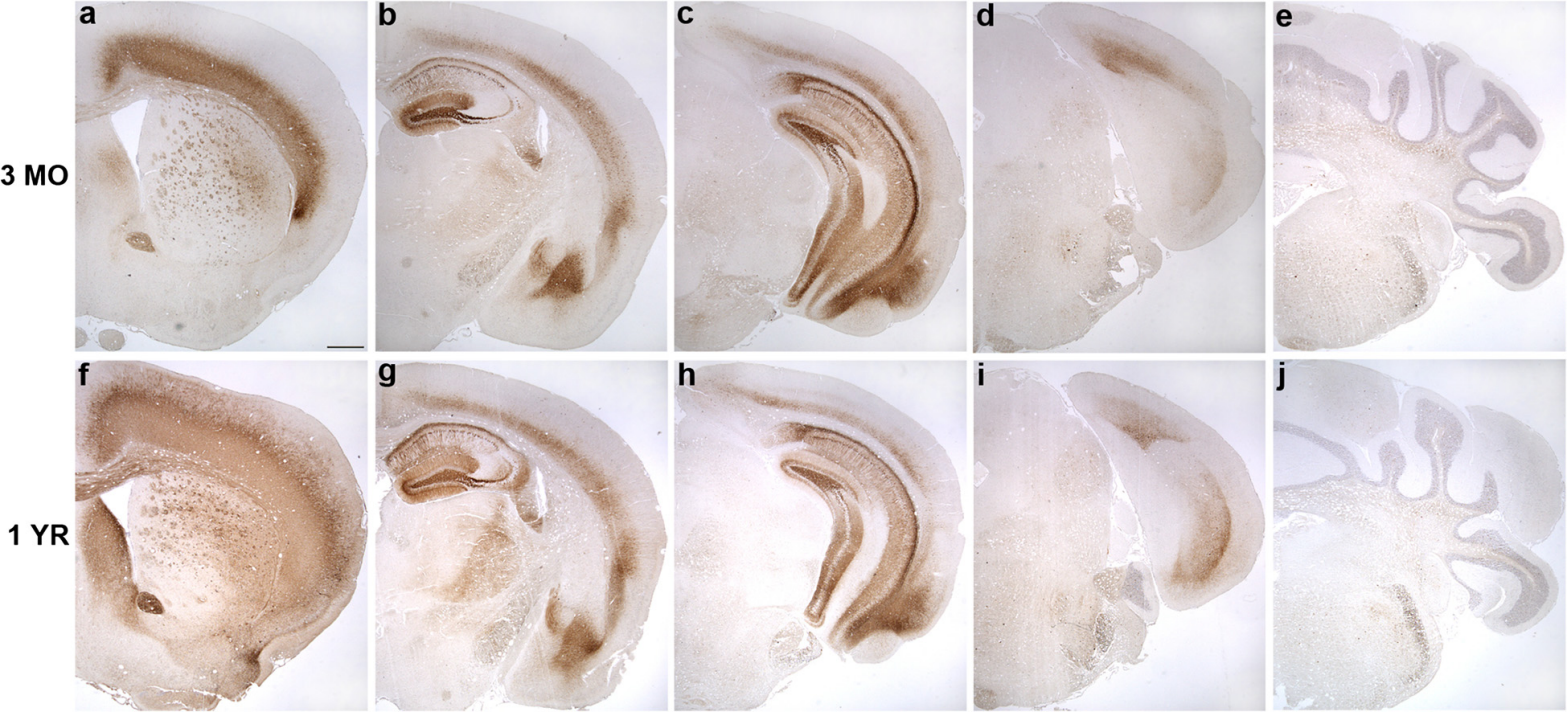
Tau4RTg2652 mice accumulate abundant MC1 immunoreactivity across multiple brain regions. At three months of age (**a**-**e**) MC1 immunoreactivity is robust in deep layers of the cortex and throughout the hippocampus and is more moderate in the hindbrain. By one year (**f**-**j**), MC1 immunostaining in the forebrain has spread to all cortical layers and throughout the striatum. Scale bars = 500um

Immunohistochemistry and histology were performed to assess neuropathological changes in tau, including hyperphosphorylated and conformationally altered tau, oligomeric tau and frank neurofibrillary tangles. As shown in Fig. 2 and Additional file 2: Figure S2, phosphorylated tau as detected by antibody AT8 was widespread and robust throughout the forebrain, including all cortical layers, striatum, anterior commissure and corpus callosum. In the cortex, AT8 immunoreactive tau was localized to neuronal cell bodies as well as the neuropil, while in the striatum, pencil fibers were highly immunoreactive. In the hippocampus, the dentate molecular layer, hilus, mossy fibers, CA1 neuronal cell bodies and apical dendrites were all robustly labeled while the CA3 was moderately so. The amygdala and entorhinal cortex were also positive for AT8 in both neuronal soma and neuropil, while in the brainstem, neurons were moderately immunoreactive. In general, rostral and hippocampal brain regions demonstrated more pathology than hindbrain regions. We did not detect any robust differences in AT8 immunoreactivity between 3 month and 1 year old animals. Other phospho-tau antibodies, including AT180, AT270, and PHF1 similarly labeled neurons in the forebrain, amygdala, hippocampus and brainstem (Table 1; Fig. 2). Conformationally altered pathological tau detected by antibody MC1 was similarly present in these same brain regions, although at three months of age, MC1 cortical expression was limited to the deeper layers of the cortex. However, by one year, MC1 immunoreactivity had spread to all layers of the frontal cortex and was also more widespread in the striatum (Fig. 3).

**Table 1:** Additional phospho-tau epitopes and NFT analysis in Tau4RTg2652 mice

Antibody/Stain	F Ctx	Amy	CA1	CA3	MF	En Ctx	BS	Cb
AT180	+	++	++	+	-	+	+	-
AT270	+	+	+	+	+	+	+	+
PHF1	+	+	+	+	+	-	+	+
Thioflavin-S	-	-	-	-	-	-	-	-
Gallyas	-	-	-	-	-	-	-	-

*Abbreviations*: *F Ctx* frontal cortex, *Amy* amygdala, *MF* mossy fibers, *En Ctx* entorhinal cortex, *BS* brain stem, *Cb* cerebellum, ++ Robust, + Moderate,- Undetectable

**Fig. 4:**
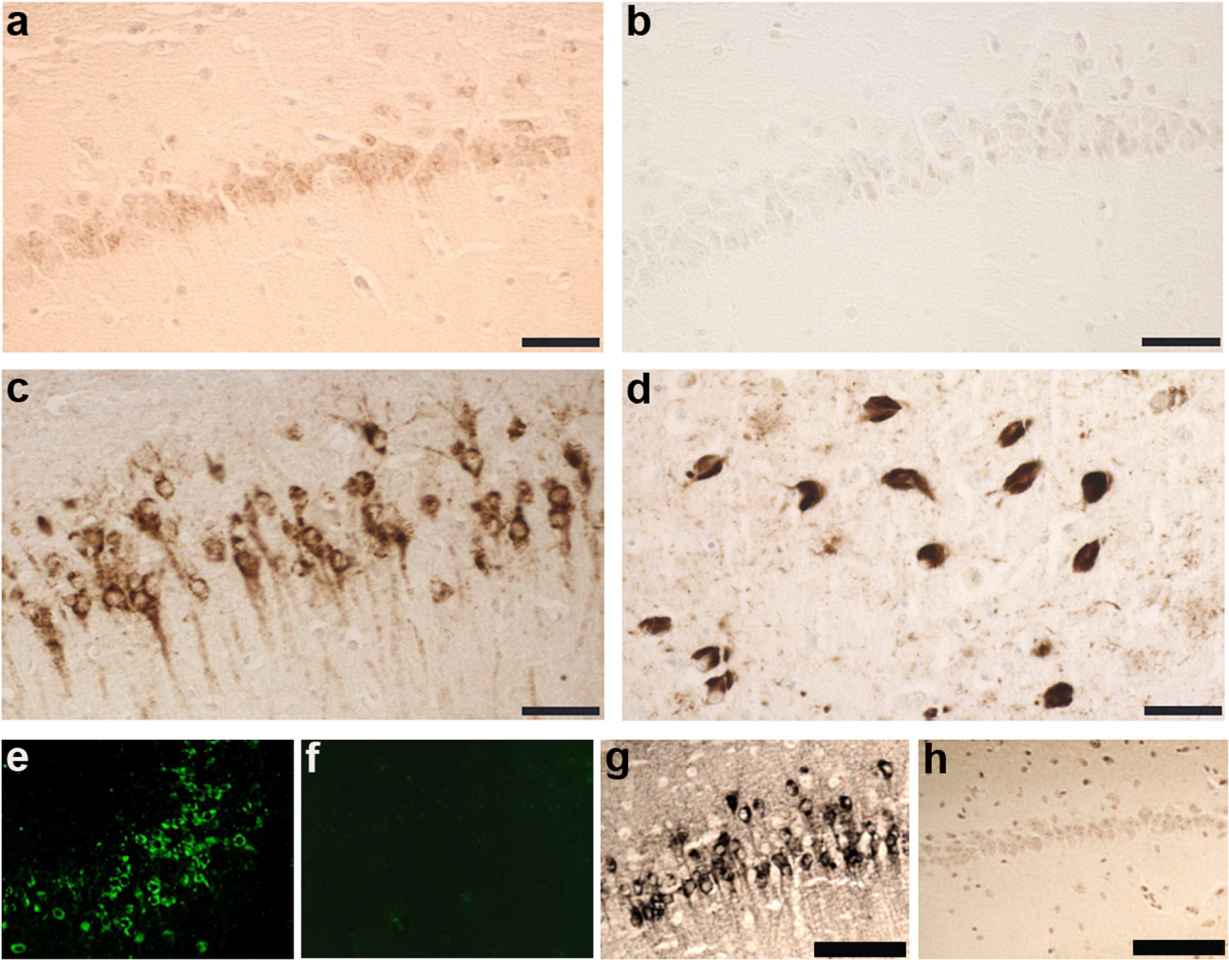
Tau4RTg2652 mice lack oligomeric or fibrillar tau species. Oligomeric tau as detected by antibody TOC1, was undetectable in most brain regions of one year old Tau4RTg2652 mice, with the exception of the hippocampal CA1 region, which demonstrated very mild immunoreactivity (**a**) compared to non-Tg mice (**b**). Hippocampal CA1 TOC1 immunoreactivity is much more robust in another tau Tg mouse model, PS19 (**c**) and in AD hippocampus (**d**). Mature neurofibrillary tangles were also lacking as evidenced by negative Thioflavin-S (**f**) and Gallyas silver stain (**h**) in aged Tau4RTg2652 mice compared to another tau Tg mouse model, PS19 (**e**,**g**). Scale bar = 50um (a-d) and 100 um (**g**,**h**)

**Fig. 5:**
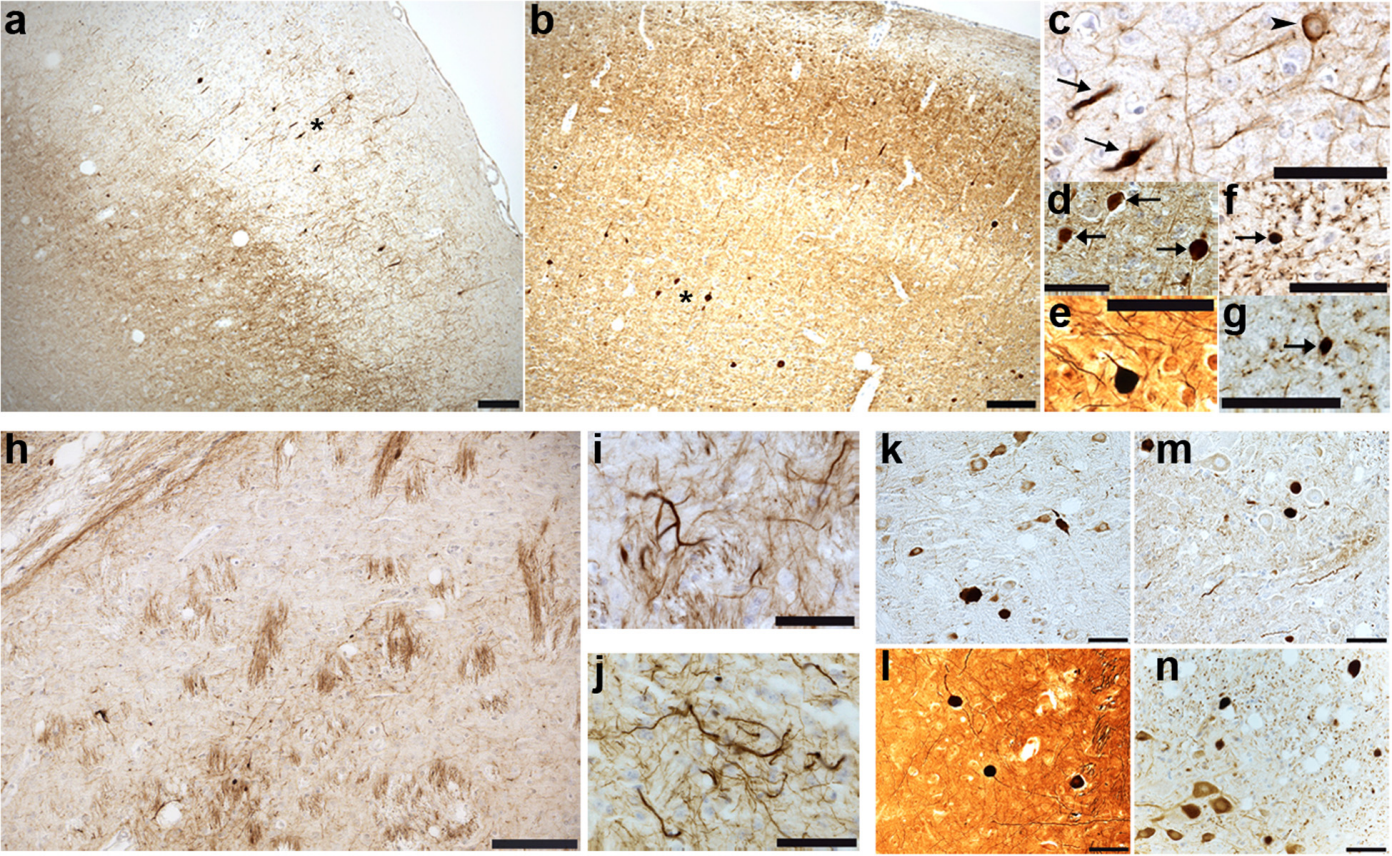
Abnormal axonal pathology, dystrophic neurites and neuritic processes are prominent features of Tau4RTg2652 mice. Grossly dilated axons resembling axonal spheroids are abundant in the frontal cortex and are immunoreactive for MC1 (**a**) and the neurofilament marker SMI31 (**b**). (**c**,**d**) High power images of regions in a and b (designated by asterisk) demonstrating the dilated axons (arrows) and a neuronal cell body (arrowhead) immunoreactive for MC1 (**c**) and SMI31 (**d**). Abnormal axonal spheroids are also detectable by Bielschowsky silver stain (**e**). Neuritic processes (dot-like pattern of staining) and axonal spheroids (arrows) are immunoreactive for AT8 (**f**) and Alz50 (**g**) in the piriform cortex. One year old Tau4RTg2652 mice exhibit robust striatal tau pathology; MC1 immunoreactivity is visible in the white fiber tracts (pencil fibers) in the striatum (**h**). Dystrophic neurites in the striatum are immunoreactive for MC1 (**i**) and AT8 (**j**). Grossly dilated axons and dystrophic neurites are detectable in the pons (**k**,**l**) and spinal cord (**m**,**n**) and are immunoreactive for AT8 (**k**,**n**), MC1 (**m**) and are Bielschowsky silver stain positive (**l**). Scale bars = 100um (**a**,**b**,**h**) and 50um (**c**-**g** and **i**-**n**)

To determine if oligomeric tau was a pathological feature of our mouse model, we immunostained with the antibody TOC1 (tau oligomeric complex 1), which selectively labels tau dimers and oligomers, but does not label filaments [13, 19, 20]. Compared to non-Tg mice, Tau4RTg2562 mice displayed mild TOC1 immunoreactivity in the CA1 region of the hippocampus (Fig. 4a,b), but TOC1 levels were nowhere near as robust as in another tau Tg mouse line (PS19) or in human AD hippocampus (Fig. 4, c and d, respectively). All other regions examined (cortex, striatum, hindbrain and spinal cord) were negative for TOC1 immunoreactivity (data not shown). Likewise, lack of Thioflavin-S fluorescence coupled with negative Gallyas silver stains (Fig. 4 e-h and Table 1) suggest very limited tau fibrillization and an absence of neurofibrillary tangles in any brain region in this mouse model. Even at extreme old age (24 months), no fibrillar tau could be detected by sliver staining (data not shown). Despite the relative lack of oligomeric or fibrillar tau species, a prominent feature of this mouse model is the presence of grossly dilated axons that are prevalent in the spinal cord, hindbrain and cortex and also detectable in the striatum. These spheroids are immunoreactive for pathological tau (MC1, AT8, and Alz50), and positively stained with the antibody SMI31 as well as the Bielschowsky silver stain, confirming their axonal nature (Figs. 5). Dystrophic neurites and abnormal neuritic processes were also detectable throughout the brain and were especially prevalent in the striatum and cortex (Fig. 5).

### Characterization of behavioral deficits

At weaning Tau4RTg2652 animals weigh significantly less than their non-Tg littermates (supplemental data, Additional file 2: Figure S2). There is a significant main effect of genotype on body weight at 3, 6, and 11 months of age (p < 0.001 at all ages in both sexes). Examination of muscle tissue from Tg mice revealed atrophic fibers indicative of neurogenic muscle atrophy, whereas no such muscle wasting was observed in non-Tg littermates (supplemental data, Additional file 2: Figure S2). This atrophy likely accounts for the weight and strength differences between the genotypes.

We used two measures to assess muscle strength in Tau4RTg2652 animals. In the grid hang test, which measures the amount of time an animal can hang onto an inverted wire cage top, we found that Tau4RTg2652 animals hang onto the inverted grid for less time at all three ages tested (main effect of genotype p < 0.001 at all ages, supplemental data). At 3 months of age, there was also a significant main effect of sex on grid hang times (*p* = 0.017). However, both male and female Tg animals had lower grid hang times than their non-Tg littermates (p < 0.001 for both sexes). The second measure of muscle strength uses a force meter to assess grip strength directly (supplemental data). At all three ages tested, Tau4RTg2652 animals had weaker forelimb grip strength (main effect of genotype p < 0.001 at all ages). As for the grid hang test, we found a significant main effect of sex on forelimb grip strength at 3 months of age (*p* = 0.006), but comparisons showed that both male (p < 0.001) and female (*p* = 0.001) Tg animals had significantly lower grip strength than non-Tg animals.

**Fig. 6:**
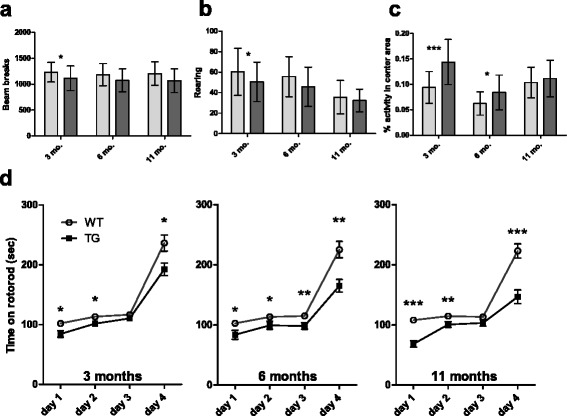
Tau4RTg2652 animals exhibit mild alterations in behavior and motor abilities. (**a**) Tau4RTg2652 animals exhibit lower activity in the open field at 3 months. (**b**) Tau4RTg2652 animals rear more often than WT in the open field at 3 months. (**c**) Tau4RTg2652 animals are more active in the center area of the open field at both 3 and 6 months. WT animals shown as light grey bars, Tau4RTg2652 animals shown as dark grey bars. All graphs show the mean for each group, with both sexes and all testing groups pooled. Error bars show the standard deviation. For main effect of genotype at each age tested: *: p < 0.05 **: p < 0.01 ***p < 0.001. (**d**) Data shown are the mean of each animal’s daily average rotorod performance (3 trials per day). Error bars show the standard error of the mean. WT animals shown as open circles, Tau4RTg2652 animals shown as solid squares

Despite the large differences in grip strength between genotypes, there were only subtle variable differences in the open field behavior of Tau4RTg2652 animals (Fig. 6a and b). At 3 months, Tg animals were hypoactive as measured by total locomotor activity recorded as photobeam breaks (main effect of genotype *p* = 0.027) or by vertical rearing behavior (main effect of genotype *p* = 0.037). However, there was also a significant main effect of sex on both measures. Comparisons revealed that the decreased activity was only significant in males, but not in females (locomotor activity: males *p* = 0.025, females *p* = 0.359; rearing: males *p* = 0.028, females *p* = 0.432). There were no significant differences in activity levels at 6 or 11 months. Overall, the decrease in spontaneous activity is relatively mild, indicating that Tau4RTg2652 animals do not have severe impairments in locomotor abilities. Thus, cognitive testing dependent on normal locomotion can be performed, as shown below. Tau4RTg2652 animals spent more time in the center area of the open field, as measured by the percent of activity that occurred in the center area (Fig. 6c), at both 3 months (p < 0.001) and at 6 months (*p* = 0.023) but not at 11 months. Time spent in the center of the open field is often interpreted as a measure of anxiety levels, or disinhibition in response to a novel environment.

Tau4RTg2652 animals displayed somewhat lower levels of motor coordination as measured by ability to walk on an accelerating rotorod (Fig. 6d). There was a main effect of genotype on rotorod times in 3 month old animals (*p* = 0.002), and planned comparisons revealed significant genotype effects on days 1, 2, and 4 of testing. In contrast to the results for open field activity at 3 months, there were no observed sex differences in rotorod abilities at 3 months. Six month old Tg animals also had decreased ability to perform on the rotorod (main effect of genotype p < 0.001), with significant effects on all days of testing. Similarly, at 11 months Tg animals performed more poorly than non-Tg (main effect of genotype p < 0.001), and planned comparisons showed significant differences on days 1, 2, and 4 of testing.

**Fig. 7:**
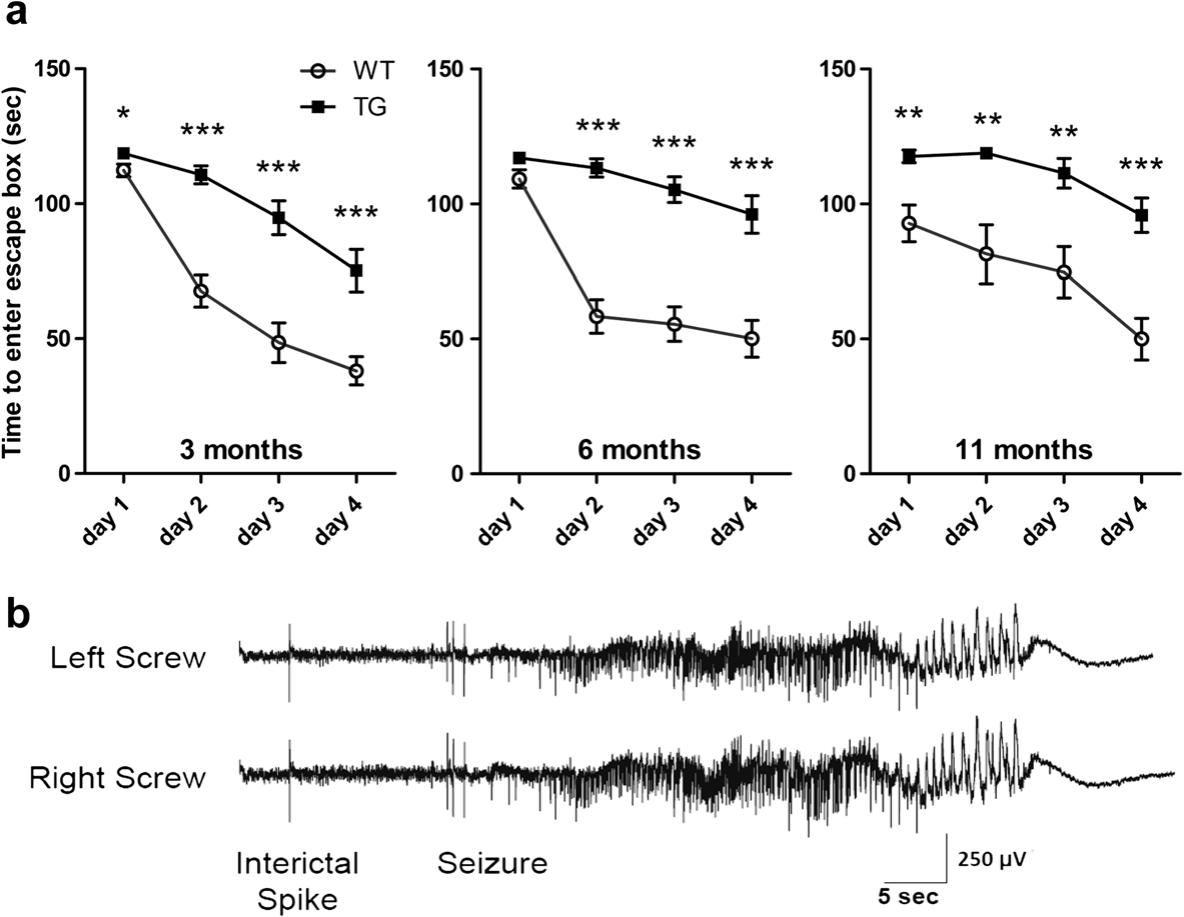
Tau4RTg2652 animals have impaired spatial memory abilities. (**a**) Tau4RTg2652 mice take longer to locate and enter the escape box on all days of testing at all ages. Data shown are the mean of each animal’s daily average performance in the Barnes maze (2 trials per day). Error bars show the standard error of the mean. WT animals shown as open circles, Tau4RTg2652 animals shown as solid squares. For planned comparisons of genotypes on each testing day: *: p < 0.05 **: p < 0.01 ***p < 0.001 (**b**) Spontaneous interictal spike and seizure recorded in bilateral screw electrodes from one of the homozygous mice studied. The electrographic seizures recorded were correlated with the behavioral seizures witnessed via the video-EEG recordings

The Barnes maze was developed to assess spatial learning and memory in aging rodents [21]. The test has previously been used successfully to measure cognitive abilities in Tg models of AD (eg, [22]), and it may be preferred to the Morris water maze since it is a less stressful test for mice [23]. Although Tau4RTg2652 animals have reduced grip strength, their spontaneous locomotion in the open field is only mildly reduced at some time points; thus we believe Barnes maze testing should provide a valid measure of cognitive abilities in these animals. Barnes maze testing revealed cognitive deficits in Tau4RTg2652 animals at all ages tested (Fig. 7a). There was a main effect of genotype on time to enter the escape box in all three age groups (p < 0.001 for each). At 3 months, planned comparisons showed significant differences between the genotypes on all four testing days. No significant sex effects were observed. Despite the observation that 3 month old male (but not female) Tau4RTg2652 animals have reduced activity levels in the open field, both sexes performed equally poorly in Barnes testing. At 6 months, the genotypes were significantly different on days 2, 3, and 4. At 11 months, Tau4RTg2652 animals were significantly slower on all four testing days.

We also measured transgene copy number in the hemizygous animals used for behavioral assays. There was no correlation between transgene copy number and severity of behavioral deficits for any of the tests performed at any age (see Additional file 1: Figure S1). However, there is a dose dependency of the transgenic phenotype as animals homozygous for the Tau4RTg2652 transgene exhibit reduced body weight, exaggerated motor abnormalities, reduced fertility, and premature death in addition to the neuropathological changes documented for the hemizygous animals. A number of the homozygous mice were also noted to have seizure-like behaviors. In order to confirm that these behavioral events were electrographic seizures, EEGs were performed on a small subset of the Tau4RTg2652 mice. The EEGs documented both interictal spike discharges and recurrent seizures in Tau4RTg2652 mice (Fig. 7b).

## Discussion

We have generated a new mouse model of tauopathy whereby pan-neuronal overexpression of the most abundant WT adult brain isoform of human tau results in the early signs of tauopathy. By 3 months of age Tau4RTg2652 mice exhibit motor and learning deficits accompanied by extensive accumulation of soluble human tau and measurable detergent insoluble aggregated tau species. In particular, Tg mice were found to have weaker muscle strength as early as 3 months of age. They also exhibited decreased motor learning abilities during rotorod training, as well as decreased maximum motor skills on the rotorod. Barnes maze testing indicated that Tg mice have deficits in spatial learning and memory abilities. Neuropathological examination revealed robust accumulation of pre-tangle and hyperphosphorylated tau in the neurons of the cortex, hippocampus, amygdala, brainstem and spinal cord as well as white matter tracts in the striatum, corpus callosum and anterior commissure. Pathological tau was evident both in the axons and somatodendritic compartment of these affected cells. The initial tempo of phosphorylated and misfolded pathological tau accumulation is rapid reaching maximal levels by three months of age, in correspondence with behavioral impairment. However, beyond 3 months there is little evidence of additional progression in tauopathy, but rather persistent maintenance of both behavioral and neuropathological phenotypes. There was no evidence for either focal or widespread neuronal loss, but neuronal cortical dysfunction was clearly evidenced by behavioral abnormalities.

One distinguishing feature of the Tau4RTg2652 model is a relatively high level of tau expression which provokes an early tauopathy-like cascade. Tau undergoes somatodendritic relocalization, becomes hyperphosphorylated and freed from microtubules, and then conformationally altered to adopt the “paperclip fold” as indicated by MC1 immunoreactivity [24, 25]. Interestingly, despite high levels of hyperphosphorylated and pre-tangle tau accumulation there is extremely limited accumulation of oligomeric tau (as detected by TOC1 antibody) [19]. Likewise the model exhibits a complete lack of Gallyas silver or thioflavin S positivity demonstrating a clear absence of tau fibrillization or NFT formation. We attribute the relative lack of progression in behavioral phenotypes after 3 months of age to a dearth of oligomeric or fibrillar tau pathological species. We propose that phospho-tau and/or “paperclip fold” conformations of tau, which occur in abundance in even young Tau4RTg2652, drive cognitive dysfunction.

Other mouse models of tauopathy driven by WT human tau transgenes have been produced previously. The ALZ17 [26] and htau40 [27] lines have several features in common, including the presence of phosphorylated and insoluble tau species in the cortex and spinal cord. Both lines express 1N4R tau under a Thy-1 promoter, similar to Tau4RTg2652. In all three cases, the resulting pathology has an early onset, observable in young adult animals, and exhibiting very slow progression if any at all. Similarly, none of the three lines have neurofibrillary tangles or any significant neurodegeneration even in old age. One important difference between these previously generated tau Tg lines and Tau4RTg2652 mice is the more widespread presence of pathological tau species in Tau4RTg2652 brains, including striatal pathology. In addition, due to the relatively mild reduction of spontaneous locomotor activity in Tau4RTg2652 mice, we can confidently interpret their impairment in the Barnes maze as a cognitive deficit. The ability to assess cognitive abilities provides additional validity and utility to Tau4RTg2652 as a model for use in future preclinical drug studies.

Models that express the 3R isoform of human tau have also been studied. Whereas humans express approximately equal amounts of 3R and 4R tau in the adult brain, adult mice normally only express 4R tau. Ishihara et al. produced Tg mice that express 3R human tau using the PrP promoter [14]. These mice exhibit markers of tau pathology (various phospho-tau epitopes and Alz50) in spinal cord as early as 1 month of age, and milder pathology in cortex by 6 months. In addition there is progressive accumulation of insoluble tau and a more severe motor phenotype than that observed in Tau4RTg2652 mice. Htau mice [28, 29] express all isoforms of human tau, but do not express any mouse 4R tau, thus producing an imbalance of 3R:4R tau. These mice have more developed tau pathology, including the presence of NFT’s and progressive neurodegeneration. Htau mice also have cognitive deficits in the absence of any observable motor dysfunction [30]. Despite the presence of mouse 4R tau and the absence of human 3R tau in Tau4RTg2652 mice, we similarly see cognitive deficits in the absence of severe motor dysfunction. Tau4RTg2652 mice provide the benefit of an early and consistent cognitive deficit starting at 3 months, as opposed to htau mice which are not distinguished from WT at 4 months. Thus Tau4RTg2652 mice may provide an alternative model for examining tau interventions without the need to age animals for extended periods.

The presence of abundant monomeric phospho-tau and pre-tangle tau species combined with the relative lack of oligomeric or fibrillar tau species makes Tau4RTg2652 a valuable model for investigations into the mechanisms of tauopathy progression. Our model does show that gross increase in cytoplasmic tau and conversion to the pre-pathologic form detected by MC1 is insufficient to drive formation of neurofibrillary tangles. Additional unknown factors are needed for the type of aggregation that leads to tangles and neurodegeneration. The lack of disease progression beyond mild cognitive and motor impairment driven by soluble pathological tau species sets the stage for studies in the Tau4RTg2652 model to identify toxic insults promoting tau fibrillization and neurodegeneration. For example, studies of extracellular spreading or seeding of tauopathy ideally require a model where tangle formation does not normally occur, but the subunits of tangles are present (ie pre-tangle tau conformers). Likewise, the model is very well suited to investigations of genetic or environmental insults that may exacerbate tauopathy.

## Conclusion

While there have been many different Tg mouse models of tauopathy reported to date (reviewed in [31]), most of them rely upon FTLD-tau causing mutations to drive tau neuropathology and the associated behavioral phenotypes. In the Tau4RTg2652 model, unique features support its adoption for preclinical studies where tauopathy driven by WT human tau is desirable. Specifically, the Tau4RTg2652 model exhibits a rapid and highly consistent accumulation of pathological tau such that by three months of age high levels of pretangle hyperphosphorylated tau and concomitant behavioral abnormalities are readily observed with statistical significance in small group sizes. Furthermore, low variability in transgene copy number, tau accumulation, neuropathology, and behavioral abnormalities provides experimental advantages not evident in many other Tg mouse models of tauopathy. Thus we favor this model for studies exploring the progression and treatment of WT tau driven tauopathies, which represent the majority of human dementia cases. Given the early onset of phenotypes and low variability, we anticipate use of the Tau4RTg2652 model will provide significant experimental advantage when conducting preclinical therapeutic studies focused on measuring engagement of early pathological tau changes as the therapeutic target.

## Additional files

Additional file 1: Figure S1. Sample data of transgene copy number in a behavioral testing cohort of 3 month old mice. Copy number is normalized to human DNA (copy number 2). Average copy number in Tau4RTg2652 mice is 143 and ranges from 126 to 155 copies per animal. Behavioral measures do not correlate with variation in copy number (not shown). Additional file 2: Figure S2. Tau4RTg2652 animals exhibit significantly reduced weight and strength at all ages. A. Tau4RTg2652 animals weigh significantly less than WT animals at all ages. B. Tau4RTg2652 animals have decreased ability to hang from an inverted grid. C. Tau4RTg2652 animals have decreased forelimb grip strength compared to WT. WT animals shown as light grey bars, Tau4RTg2652 animals shown as dark grey bars. All graphs show the mean for each group, with both sexes and all testing groups pooled. Error bars show the standard deviation. For main effect of genotype at each age tested: *: p < 0.05 **: p < 0.01 ***p < 0.001. D. Photomicrograph of section gastrocnemius muscle from Tau4RTg2652 mouse, containing normal muscle fibers (upper left side of image) and groups of small atrophic fibers suggestive of neurogenic atrophy (middle/right side of image). H&E stain; bar = 100 um. E. Photomicrograph of section of gastrocnemius muscle from non-Tg control mouse. H&E stain; bar = 100 um.
