# Supplementary figures and images for: High copy wildtype human 1N4R tau expression promotes early pathological tauopathy accompanied by cognitive deficits without progressive neurofibrillary degeneration
Source: Acta Neuropathol Commun. 2015 Jun 4;3:33. doi: 10.1186/s40478-015-0210-6 (PMC4453289; doi:10.1186/s40478-015-0210-6)

human Tau Copy Number

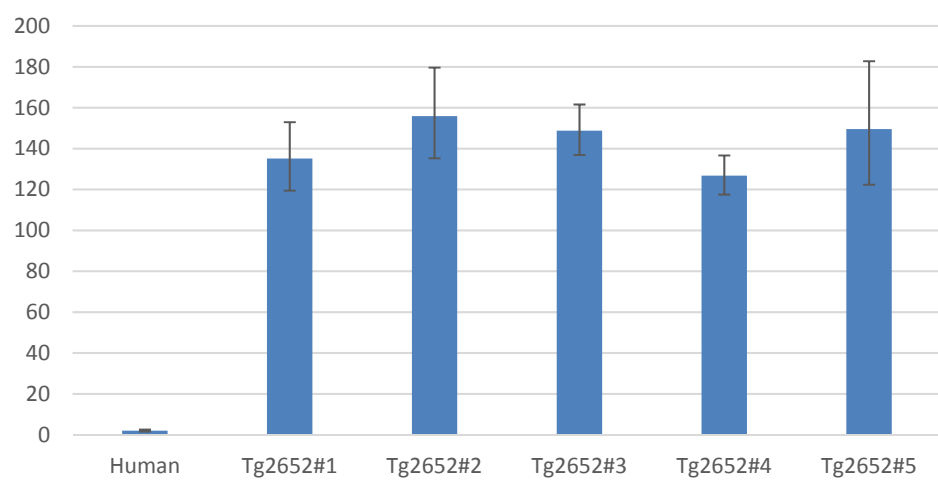

Supplement: Additional file 1: Figure S1. — Sample data of transgene copy number in a behavioral testing cohort of 3 month old mice. Copy number is normalized to human DNA (copy number 2). Average copy number in Tau4RTg2652 mice is 143 and ranges from 126 to 155 copies per animal. Behavioral measures do not correlate with variation in copy number (not shown). [file 40478_2015_210_MOESM1_ESM.pdf]

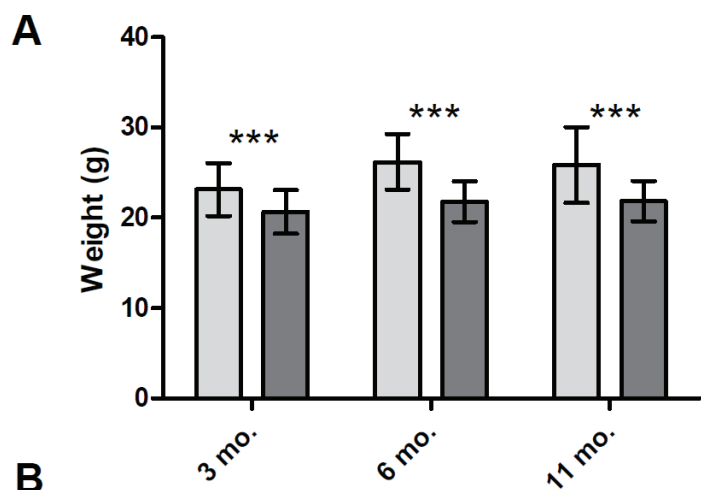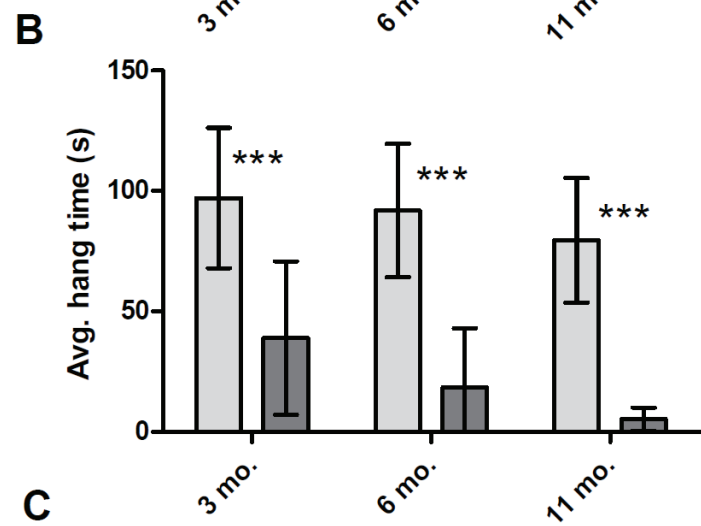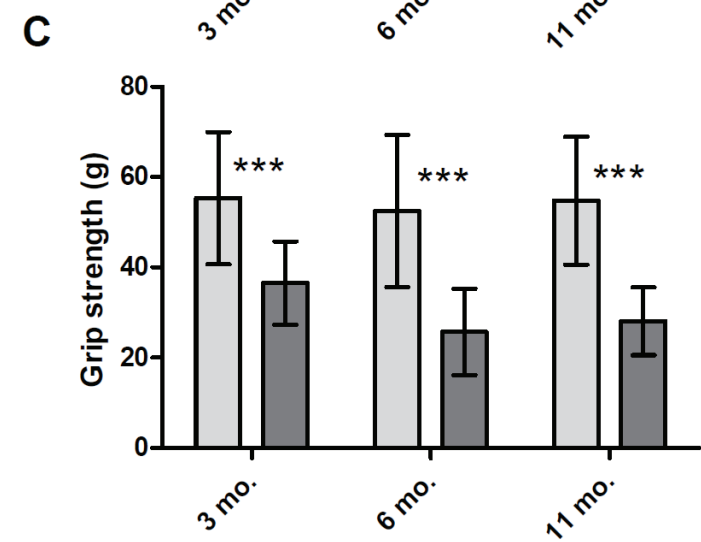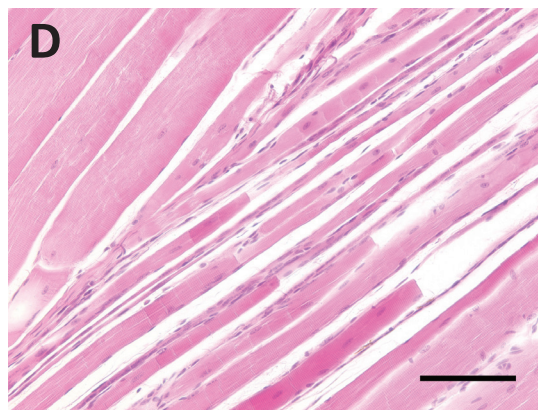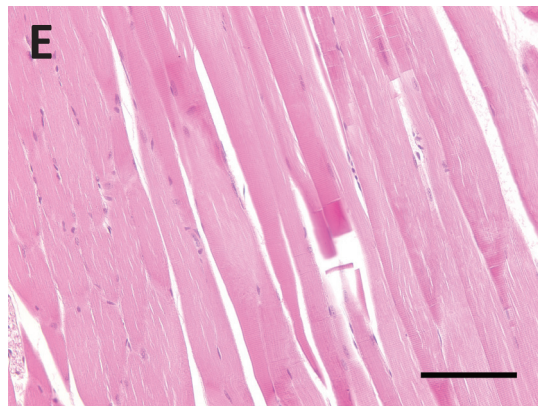

Supplement: Additional file 2: Figure S2. — Tau4RTg2652 animals exhibit significantly reduced weight and strength at all ages. A. Tau4RTg2652 animals weigh significantly less than WT animals at all ages. B. Tau4RTg2652 animals have decreased ability to hang from an inverted grid. C. Tau4RTg2652 animals have decreased forelimb grip strength compared to WT. WT animals shown as light grey bars, Tau4RTg2652 animals shown as dark grey bars. All graphs show the mean for each group, with both sexes and all testing groups pooled. Error bars show the standard deviation. For main effect of genotype at each age tested: *: p < 0.05 **: p < 0.01 ***p < 0.001. D. Photomicrograph of section gastrocnemius muscle from Tau4RTg2652 mouse, containing normal muscle fibers (upper left side of image) and groups of small atrophic fibers suggestive of neurogenic atrophy (middle/right side of image). H&E stain; bar = 100 um. E. Photomicrograph of section of gastrocnemius muscle from non-Tg control mouse. H&E stain; bar = 100 um. [file 40478_2015_210_MOESM2_ESM.pdf]
